# A flexible capacitive photoreceptor for the biomimetic retina

**DOI:** 10.1038/s41377-021-00686-4

**Published:** 2022-01-01

**Authors:** Mani Teja Vijjapu, Mohammed E. Fouda, Agamyrat Agambayev, Chun Hong Kang, Chun-Ho Lin, Boon S. Ooi, Jr-Hau He, Ahmed M. Eltawil, Khaled N. Salama

**Affiliations:** 1 Sensors lab, Advanced Membranes and Porous Materials Center, Computer, Electrical and Mathematical Science and Engineering Division, King Abdullah University of Science and Technology (KAUST), Thuwal, 23955-6900 Kingdom of Saudi Arabia; 2 Communication and Computing Systems Lab, Computer, Electrical and Mathematical Science and Engineering Division, King Abdullah University of Science and Technology (KAUST), Thuwal, 23955-6900 Kingdom of Saudi Arabia; 3 Department of Electrical Engineering and Computer Science, University of California-Irvine, Irvine, CA 92612 USA; 4 Department of Electrical, Computer and Energy Engineering, Arizona State University, Tempe, AZ USA; 5 Computer, Electrical and Mathematical Science and Engineering Division, King Abdullah University of Science and Technology (KAUST), Thuwal, 23955-6900 Kingdom of Saudi Arabia; 6 Department of Materials Science and Engineering, City University of Hong Kong, Hong Kong SAR, China

**Keywords:** Polymers, Photonic devices

## Abstract

Neuromorphic vision sensors have been extremely beneficial in developing energy-efficient intelligent systems for robotics and privacy-preserving security applications. There is a dire need for devices to mimic the retina’s photoreceptors that encode the light illumination into a sequence of spikes to develop such sensors. Herein, we develop a hybrid perovskite-based flexible photoreceptor whose capacitance changes proportionally to the light intensity mimicking the retina’s rod cells, paving the way for developing an efficient artificial retina network. The proposed device constitutes a hybrid nanocomposite of perovskites (methyl-ammonium lead bromide) and the ferroelectric terpolymer (polyvinylidene fluoride trifluoroethylene-chlorofluoroethylene). A metal-insulator-metal type capacitor with the prepared composite exhibits the unique and photosensitive capacitive behavior at various light intensities in the visible light spectrum. The proposed photoreceptor mimics the spectral sensitivity curve of human photopic vision. The hybrid nanocomposite is stable in ambient air for 129 weeks, with no observable degradation of the composite due to the encapsulation of hybrid perovskites in the hydrophobic polymer. The functionality of the proposed photoreceptor to recognize handwritten digits (MNIST) dataset using an unsupervised trained spiking neural network with 72.05% recognition accuracy is demonstrated. This demonstration proves the potential of the proposed sensor for neuromorphic vision applications.

## Abstract (web summary)

The illustration depicts capacitive photoreceptor (CPR) mimicking the rod cell and its functionality simulation. CPRs connected to the sensing circuit followed by a spiking neural network mimics the retina functionality.

## Graphical abstract

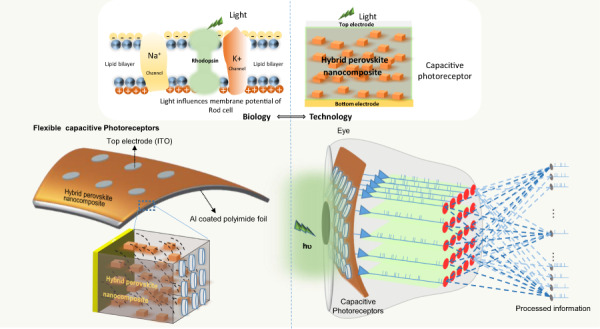

## Introduction

**Fig. 1:**
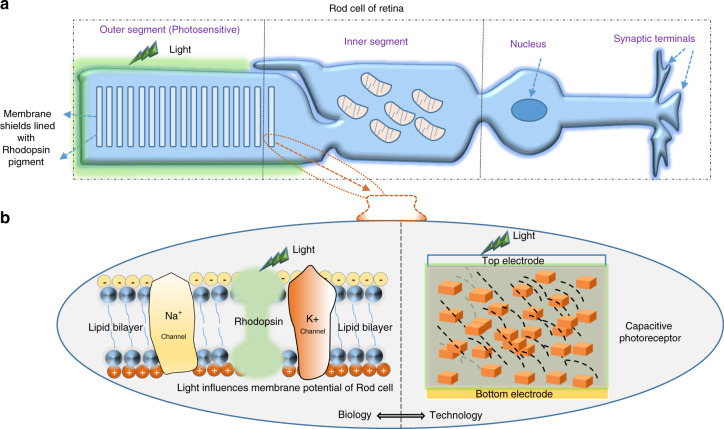
A closer look into the rod cell of mammalian’s retina. **a** Schematic of the typical Rod cell in mammalian’s retina. The rod cells absorb light in the retina and transmit signals to the brain. The outer segment of the rod cell comprises disc membranes lined with rhodopsin, which contains photosensitive pigments. **b** Illustrative comparison of rod cell membrane potential in the retina and the fabricated capacitive photoreceptor. Light induces the change in membrane potential of rod cells in the retina. The pigments in the rhodopsin absorb light and influence Na+ ion flow, which results in the hyperpolarization of the Rod cell. Similarly, light induces a change in the capacitance of the devised photoreceptors

The biomimetic microelectronic devices are indispensable for human-inspired robotics and neuromorphic computing applications^1–4^. A change in paradigm from sensing to perception aided by machine learning and deep neural networks; revolutionizing perceptive intelligence such as computer vision and voice processing. Our human brain receives most of the information (80%) through the eyesight^5^. Various hierarchical perceptive processes take place in the eye to form the vision in the brain. This includes photoreceptors’ photon reception and encoding the illumination information into varying spike frequencies of the cells through ganglion cells of the retina^6^. The photoreceptor cells, namely rod cells, and cone cells absorb the light in the retina. The rod cells’ density in the retina is much higher than the cone cells, and they are responsible for light sensing in low light conditions^7^. Whereas the cone cells are responsible for light sensing in bright conditions. The typical schematic of the rod cell is shown in Fig. 1a. The outer segment of the rod cell is photosensitive due to the presence of pigments called retinal and opsin, which are located in the rhodopsin. These pigments in the rod cell are responsible for the change in the membrane potential upon light illumination (as illustrated in Fig. 1b). The rod cell membrane undergoes hyperpolarization upon light illumination, and that results in a decrease in the spiking frequency of rod cells. Thus, photoreceptor cells encode the light information into the spike train which are transmitted to ganglion cells for further visual transduction process. The retina of the eye performs the parallel signal operation and follows the computing strategy known as computing in the sensor^8^. Hence, artificial retina networks are faster and smarter than the conventional image processing devices^9,10^.

To construct the artificial retina network, it requires a tunable artificial neuron with functionalities of photoreceptors cells and ganglion cells. In other words, the membrane potential in photoreceptor neurons is affected by the light signals in addition to the electrical signals of other neurons. The traditional integrate-and-fire neuron model for data processing doesn’t possess the functionality of photoreceptors. There are few inspiring works that perform image sensing mimicking the human eye; Zhang et al.^4^ demonstrated a hemispherical eye developed using silicon (Si) nano-membrane in the origami approach, and they have employed Si photodetectors as photoreceptors. Recently, Gu et al.^3^ demonstrated a perovskite-based hemispherical biomimetic eye for robotics and visual prosthesis applications. They have fabricated a perovskite nanowire array as photoreceptors and demonstrated image sensing using the eye. However, each pixel in these biomimetic eyes requires biasing that leads to high static power consumption since they are photodetectors and closer to the conventional image sensors. One of the popular biological vision cameras, called dynamic vision sensing (DVS) cameras, also employs photodiodes as photoreceptors^11^. The capacitors are usually used to mimic the cell membrane in CMOS-based electrical neurons^9^. Moreover, the capacitive neural networks, rather than the resistive/conductance-based approach^12–14^, featured better emulation of neural functionalities and low static power consumption^15^. Thus, there is a need for a tunable photoreceptor to develop artificial retina networks for efficient, perceptive intelligent applications.

Furthermore, there are studies on the capacitors using lead zirconate titanate (PZT) thin films that are sensitive to UV illumination^16,17^. These thin-films exhibited varying dielectric properties upon light illumination but not under visible wavelength. Such a phenomenon has been useful in developing photosensitive and photostrictive actuators^18^. There are also reports on visible light photo capacitors for the charge storage using dye-sensitized semiconducting nanoparticles^19^, phosphors^20^, photosensitive conjugated polymers^21^, and through a hybrid plasmonic effect of Ag nanowires^22^. However, hybrid perovskites^23,24^ attracted considerable attention because of their exceptional optoelectronic properties such as excellent light absorption, long carrier lifetime, low trap density, giant optical anisotropy^25^, and high carrier mobility^26–28^. Thanks to these properties, higher photocurrents, and higher quantum efficiencies were observed in perovskite-based optoelectronic devices, namely; solar cells^29–32^, photodetectors^33–35^, photo-transistors^36^, and devices for various applications like photo-sensing^37^, lasing^38,39^ and emission^40^. The hybrid perovskites are sensitive to moisture and oxygen, resulting in degradation of the device performance, which is the major hurdle for the commercialization of perovskite devices^23,34^. On the other hand, polyvinylidene fluoride (PVDF) based ferroelectric polymers have an electroactive phase and are widely used as a dielectric medium in various energy storage applications^41^, fractional-order capacitors^42,43^, optoelectronic devices^44^, and plastic solar cells^45^. Especially, a terpolymer of PVDF such as PVDF-TrFE-CFE exhibits a high dielectric constant (high *k* value), and low dielectric losses^46^, which can offer high capacitance and high dynamic range in the tunability of capacitors. Moreover, it is a relaxor ferroelectric polymer, high charging and discharging efficiencies are achievable for capacitive energy storage applications^47,48^. The choice of flexible substrates and the design of interconnects on flexible substrates for wearable electronics is crucial. One of the promising and commercially available substrates is Kapton (polyimide), and the design of interconnects on this substrate is also well known. Hence, Kapton substrate is the appropriate choice to explore flexible sensors and circuit architectures^49^. Moreover, it provides an opportunity to develop bio-compatible systems and implement telecommunication protocols^50^ between flexible sensors and flexible peripheral electronics.

Herein, we demonstrate the light intensity capacitive photoreceptor (CPR) that mimics the retina’s rod cells. The capacitance of CPRs is dependent on visible light illumination and can lead to the development of the artificial retina by integrating with peripheral electronics^9^. To fabricate CPRs with excellent light tunable properties, we require materials that are photosensitive and materials that have tendencies to tune the dielectric properties. In order to obtain the combination of exceptional optoelectronic and ferroelectric properties, we prepared a hybrid composite of methylammonium lead bromide perovskite (MAPbBr_3_) and the terpolymer polyvinylidene fluoride trifluoroethylene-chlorofluoroethylene (PVDF-TrFE-CFE). We demonstrated the fabrication and characterization of flexible CPR, which has a frequency-dependent capacitance within the range of 1−100 kHz. The hybrid perovskites as fillers in the ferroelectric polymer attribute to modulate the dielectric properties proportional to the intensity and wavelength of the incident light. The capacitive change with respect to the wavelength of the incident light mimics the spectral sensitivity curve of human photopic vision with the maximum response in the greenish-yellow regime. The photoresponse of these CPRs is reproducible with negligible hysteresis. Furthermore, the fabricated device is resistive to humidity and oxygen due to the encapsulation of the hybrid perovskites in the hydrophobic ferroelectric terpolymer (FP). To the best of our knowledge, we report the longest stability measurement of hybrid perovskites (~129 weeks) owing to their PVDF-TrFE-CFE encapsulation. The proposed device is modeled with an RC network and integrated with a novel low power spike oscillator to generate the spike train with a firing rate proportional to the incident light intensity and wavelength (color). Then, the functionality of the proposed CPR and sensing circuit is demonstrated through simulation to recognize the handwritten digits (MNIST) dataset using an unsupervised trained spiking neural network.

## Results

**Fig. 2:**
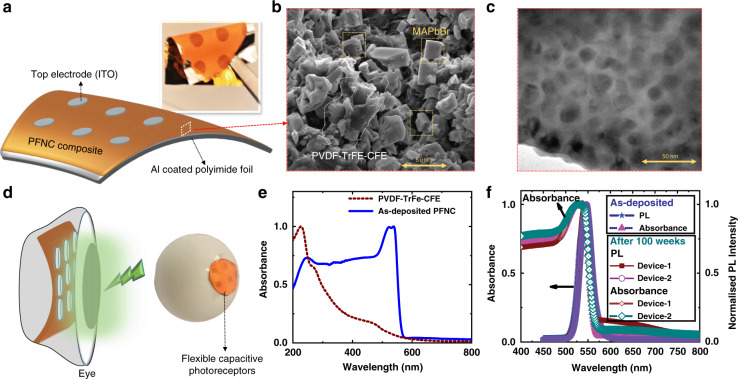
The flexible capacitive photoreceptors (CPRs) and material characterization. **a** Schematic of the flexible CPR showing the perovskite ferroelectric nanocomposite (PFNC) sandwiched between transparent top electrodes indium tin oxide (ITO) and aluminum (Al) coated polyimide substrate. (Inset shows the fabricated device). **b** The cross-sectional scanning electron microscope (SEM) image of the PFNC thin-film. **c** Transmission electron microscope image of the PFNC showing the perovskite nanocrystals embedded in the polymer. **d** Representative schematic to depict that flexible capacitor as photoreceptors of the retina and the digital photograph of the flexible CPRs array shaped into hemispherical form. **e** UV−VIS absorbance spectra of perovskite ferroelectric polymer nanocomposite (PFNC) and the ferroelectric polymer. **f** Absorbance and photoluminescence spectra of the as-deposited PFNC and devices after 100 weeks of fabrication, confirming the stability of the CH_3_NH_3_PbBr_3_ in the nanocomposite

Facile process flow was adopted for the fabrication of flexible CPRs. The perovskite ferroelectric nanocomposite (PFNC) was sandwiched between metallic electrodes (Fig. 1b), where the top electrodes were transparent and fabricated an array of metal-insulator-metal (MIM) capacitors, and they are being called CPRs. The schematic of the fabricated CPRs on a flexible substrate in the MIM configuration is shown in Fig. 2a and the inset digital photograph depict the flexibility of the fabricated devices. Figure 2b shows the cross-sectional scanning electron microscopic (SEM) image of the PFNC thin-film showing the perovskite cubic crystals intermixed within the layers of the PVDF-TrFe-CFE polymer. The transmission electron microscopic image (TEM) of the PFNC depicts the MAPbBr_3_ nanocrystals embedded in the FP (Fig. 2c). The X-ray diffraction pattern shown in Fig. S1 indicates the purity of perovskite crystals in the composite, which was undisturbed in the polymer. The signature peaks further confirm the cubic structure of the MAPbBr_3_ perovskite crystals^51^ in PVDF-TrFE-CFE FP^52^. The promising application of flexible CPR as the retina of the eye is representatively shown in Fig. 2d. Thanks to its flexible properties, the fabricated device can be shaped into any desirable shape using simple origami techniques. For instance, we showed that it could be shaped into hemispherical to mimic the eye (Fig. 2d). Note that flexible CPRs are not bio-compatible since the proposed composite constitutes heavy metal Pb. Hence, for bio-compatible bio-mimetic eye further research employing Pb, less perovskites have to be carried out at the cost of the optical performance^53^.

### Photosensitivity of the CPR

The UV−VIS spectra of the as-deposited PFNC and the pure FP thin-films are shown in Fig. 2e. The PFNC composite exhibited absorbance in the UV and part of the visible regime. The pure PVDF-TrFE-CFE thin-film has peak absorbance near 200 nm, relatively weak absorbance in the visible regime (400−500 nm), and from the spectra of both samples, it can be seen that the peak near ~ 200 nm corresponds to the FP thin-film^54^. The broad absorption of PFNC thin-film until 563 nm in the visible regime is attributed to the MAPbBr_3_ nanocrystals in the composite. The PFNC composite exhibited strong photoluminescence (PL) due to the presence of MAPbBr_3_ nanocrystals in the ferroelectric polymer, as shown in PL spectra (Fig. 2f). The band-edge cutoff for the PFNC composite is ~2.2 eV (λ = 563 nm). So far, the characterization studies indicate that PFNC is light-sensitive. Therefore the fabricated devices were characterized under controlled light conditions. The device characterization and mechanism study were performed by measuring the magnitude of impedance (|Z|) and phase angle under dark and under various light intensities of multiple commercial light-emitting diodes (LEDs). From previous studies, it is known that semiconducting fillers in the ferroelectric polymers exhibit frequency-independent behavior in the frequency range of 1−100 kHz^55^; hence all the measurements performed in this work were limited to this range.

### Electrical behavior of the CPR under light illumination

**Fig. 3:**
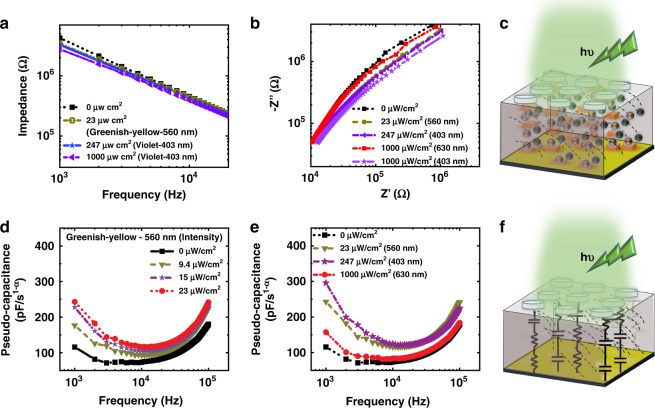
The frequency-independent response of capacitive photoreceptors (CPRs). **a** The impedance of the CPRs corresponding to the change in light intensities of various LEDs. **b** The Nyquist impedance plot of CPRs indicating the frequency-independent behavior. **c** Schematic illustration of photo-generated carriers in PFNC. Pseudo-capacitance variation **d** under the illumination of greenish-yellow LED and **e** under various LEDs of different intensities. **f** Schematic illustration of varying dielectric properties in CPR, influencing its fractional order impedance upon light illumination

The CPR with PFNC is very sensitive to light due to the presence of MAPbBr_3_ nanocrystals in the nanocomposite. The fabricated CPR was illuminated at different intensities using different commercial LEDs in the visible spectrum; violet (~ λ_peak_ = 403 nm), green (~ λ_peak_ = 520 nm), greenish-yellow (~ λ_peak_ = 560 nm), yellowish-orange (~ λ_peak_ = 590 nm), and red (~ λ_peak_ = 630 nm). The beam profile and the intensities of these LEDs were homogenized using a custom-built setup, as shown in Fig. S2a. From Fig. 3a, it is evident that even with the slight variation in the greenish-yellow LED intensity, the impedance of the CPR is decreasing significantly. The Nyquist impedance plot (Fig. 3b) for different light intensities of 3 LEDs indicates that the CPRs exhibit frequency-independent behavior. The capacitance of such devices that exhibits frequency-independent behavior is called pseudo-capacitance (*C*_α_), which was estimated from the impedance and phase angle of the device (S3, Supplementary information). The *C*_α_ of the CPRs increased with the increasing light intensity, and frequency-independent behavior at each light condition is further evident in the capacitive response shown in Fig. 3d, e.

The response of multiple CPRs illuminated to 23 μW/cm^2^ greenish-yellow light is overlapped with the response of devices illuminated to 247 μW/cm^2^ violet light, as depicted in Fig. 3a, b, d. The capacitive response is higher in the case of greenish-yellow light when compared to violet or red, which indicates that the composite is more sensitive to the greenish-yellow. The higher sensitivity in the greenish-yellow regime is due to the maximum absorbance of the composite in this regime (Fig. 2e). When devices were illuminated with the red LED even at 1000 μW/cm^2^, the change in *C*_α_ is negligible, where the absorbance of the composite is almost negligible. The observed electrical response is in-line with the discussed UV−VIS and PL spectra of the PFNC composite. The capacitive response was measured under other LEDs, and Nyquist impedance plots are shown in Figs. S3, S4, respectively. All these results together evident that the CPRs’ response is sensitive to the wavelength and intensity of the light. Similar to the photoreceptors in the retina, which induces membrane potential change upon varying light intensity^9^, the capacitance of the CPR is found to be variable with light intensities. The trend in variation of *Z* and *C*_α_ indicates that there is an increase in the conductivity of the dielectric medium^55,56^ due to the photo-generated charge carriers (Fig. 3c). The equivalent circuit representing the frequency-independent behavior can be modeled as RC ladder circuits connected in parallel and, as shown in Fig. 3f^57^. It illustrates the photosensitive impedance change in the CPR. In the dark condition, the capacitance density is ~1 nF/cm^2^. Achieving the capacitance densities comparable to the biological membrane capacitances is challenging with the existing CMOS scaling and technology^58^. The higher baseline capacitance density in the PFNC is due to the ferroelectric electric properties of the FP in the composite.

Halide perovskites are highly sensitive to polar molecules such as water and oxygen, owing to their ionic nature. This results in the phase transition of the perovskites, which eventually leads to poor optical performance^34^. Hence, the stability of these perovskites in the air ambience is poor. This degradation study of perovskites can be performed using photoluminescence (PL) spectroscopy. The phase transitions, which are the indications of degradation, result in the peak broadening or shift^59^. It is evident that even after 100 weeks of aging, there is neither significant linewidth broadening nor peak PL wavelength shift in the PL spectra (Fig. 2f) of the devices with the PFNC composite. The devices used for stability studies were kept in the petri dish and stored in the ambient air (room temperature ~23 °C and ~40% R_H_ humidity). This indicates that the composite performance is very stable, and there is no degradation of MAPbBr_**3**_ nanocrystals as they are encapsulated in the PVDF-TrFE-CFE polymer. Since PVDF polymers are more hydrophobic^60^, PFNC is more resistant to oxygen and moisture absorption. The absorbance spectra are also undisturbed, as seen in Fig. 2f, the stored devices still absorb the light similar to the as-deposited devices. Figure S5 also indicates the response measured with LED illumination after 100 weeks of storage, and the devices are responsive to the light as discussed earlier. Stability is not an issue anymore in the case of PFNC; thus, we addressed a significant shortcoming of perovskite devices. To our knowledge, these are the longest stability studies conducted on hybrid perovskite-based devices. As per reported studies^61,62^, the solar cell efficiency has been increased in perovskite solar cells with PVDF polymer as an additional layer due to the reduction in electron−hole recombination. Hence, the PFNC composite can lead to exploring a wide variety of efficient and stable optoelectronic devices.

**Fig. 4:**
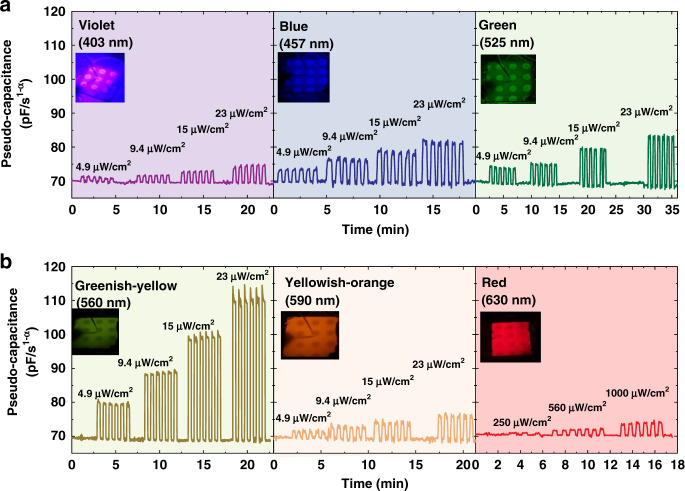
The transient response of the capacitive photoreceptors (CPRs) under the illumination of LEDs at various intensities, indicating the tunable and reproducible properties of CPRs (measured at 10 kHz). **a** violet (~ λ_peak_ = 403 nm), blue (~ λ_peak_ = 457 nm), and green (~ λ_peak_ = 525 nm) **b** greenish-yellow (~ λ_peak_ = 560 nm), yellowish-orange (~ λ_peak_ = 590 nm) and red (~ λ_peak_ = 630 nm)

**Fig. 5:**
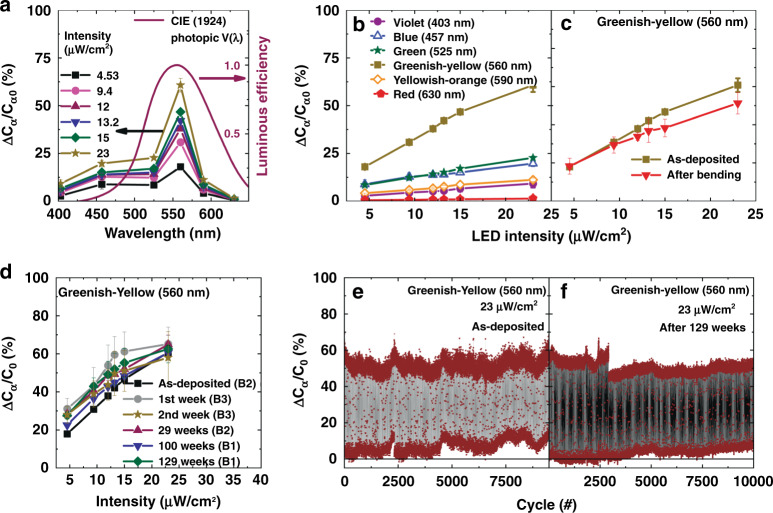
Wavelength-dependent characteristics and reliability of capacitive photoreceptors (CPRs). **a** The variation in *C*_α_ to various exciting light wavelengths, and comparison of the photo-response of the CPR with the CIE (1924) photopic luminous efficiency function of human eye (data is obtained from public database available at http://www.cvrl.org). **b** The linear response of multiple CPRs proportional to the intensity of the light, and it shows the higher sensitivity to greenish-yellow light (560 nm), **c** the response and performance of flexible CPRs without bending and after bending (bent radius ~1 cm). **d** The response of fabricated CPRs measured at various intervals after the devices’ fabrication, and the response shows the devices are stable in air at least for 129 weeks after fabrication. The devices were stored in the petri dish and in-room temperature ambience (~23 °C and 40% RH). The endurance of devices; **e** as-deposited, **f** 129 weeks after fabrication measured under the excitation of modulating LED light pulses (Greenish yellow, 560 nm, 23 mW/cm2, LED on time is 10 s and OFF time is 5 s)

The proposed CPR shows high electrical stability in the dark condition, the *C*_α_ at 10 kHz for a long time, and the negligible deviation from the baseline was observed (Fig. S6). In order to understand the transient and the repeatable behavior of the CPR upon illumination, the LEDs were modulated with voltage pulses (pulse width ~20 s) and measured the *C*_α_ of multiple devices under the illumination of all LEDs. The transient response of the CPR devices excited with multiple LEDs at various intensities is shown in Fig. 4a, b. The digital photographs of homogenized incident light beams during the characterization are depicted in the in-set. From these measurements, it can be seen that the *C*_α_ modulates according to the incident light intensity, CPR devices exhibit baseline capacitance in the dark and a step response with the light pulse. The varying intensity of LEDs is reflected in the varying *C*_α_ magnitude of CPR devices. The extent of variation in the *C*_α_ is also dependent on the exciting light wavelength. The change in *C*_α_ (Eq. 1) of CPR at various wavelengths in the visible spectrum is shown in (Fig. 5a). The CPR devices’ exhibited maximum response in the greenish-yellow regime due to the maximum absorbance of PFNC composite in this regime, as discussed earlier. Interestingly, the spectral density curve of the human eye’s photopic vision has a similar response to colors as of CPR^63^. This shows that the devised composite has a huge potential to mimic the eye. The response of CPR to different colors and intensities is linear but with varying sensitivity, as shown in Fig. 5b. The effect due to the bending of multiple CPR in the response was measured, and Fig. 5c shows that there is no significant variation in the response at lower intensities, but a small deviation at higher intensities because of bending. The incident beam and the extent of bending (bending radius ~1 cm) used during the characterization are shown in Fig. S7. We posit that a slight decrease in the response due to bending is due to a slight variation in the angle of the incident light, which might cause a slight decrease in incident light intensity.

The CPR devices were fabricated at various intervals in different batches (B1, B2, and B3), B1 is the earliest batch, and B3 is the recent batch of fabricated devices. These devices’ response was tested under homogenized conditions at various intervals to understand devices’ air stability after the storage. Interestingly, the devices (batch-B1) are stable even after 129 weeks of storage in robust ambient conditions (placed in a petri dish, ~22−23 °C and 40% RH), and the response of all devices that were tested after several weeks is comparable to as-deposited devices (Fig. 5d). This shows the remarkable ambient stability of devices. Moreover, devices also exhibited excellent endurance ~10,000 cycles under the illumination of modulating light pulses measured in ambient air. Interestingly, the endurance of the 129-week old device (Fig. 5f) is the same as of the as-deposited device (Fig. 5e) that further demonstrate the stability of the devices. The CPR responds within 20 ms, but the response saturates in 0.8 s after turning ON the LED (Fig. S6), which is also similar to the adaptation mechanism of the human eye. The devices reach the baseline in 0.92 s after the instant of turning OFF. Hence, to test the endurance of devices with the longer exposure time, the LED was programmed to ON for ~10 s and OFF for 5 s, and the capacitance was measured for every 5 s. This also shows that devices have shown stability under operation for at least 41 h.

1 $$\frac{{{\Delta}{{{C}}}_\alpha }}{{{{{C}}}_{\alpha 0}}}{{{\mathrm{\% }}}} = \frac{{{{{C}}}_\alpha ({{{\mathrm{under}}}}\;{{{\mathrm{light}}}}) - {{{C}}}_{\alpha 0}({{{\mathrm{under}}}}\;{{{\mathrm{dark}}}})}}{{{{{C}}}_{\alpha 0}({{{\mathrm{under}}}}\;{{{\mathrm{dark}}}})}} \ast 100$$

### Operation mechanism of CPR

**Fig. 6:**
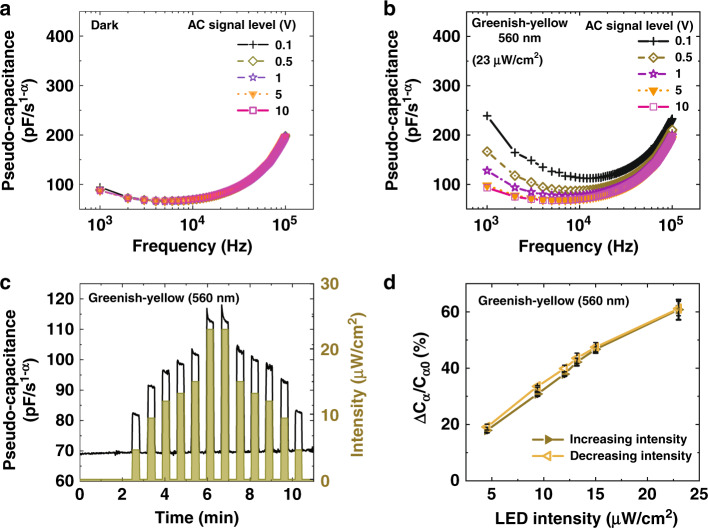
Mechanism and memory effect study of capacitive photoreceptors (CPRs). The variation in the capacitance due to the AC signal level perturbations to study the effect of the photo-generated carriers in the conductivity modulation of the dielectric **a** under dark, **b** under greenish-yellow light. **c** The transient response of the CPRs (at 10 kHz) with increasing and the subsequent decreasing greenish-yellow light intensities, **d** the corresponding response of CPRs with the negligible charge accumulation and hysteresis

The metal halide perovskites are well known for their mixed electronic and ionic conduction mechanisms^64,65^. The generation of non-equilibrium charge carriers upon light illumination is affecting the dielectric properties. To further understand the effect of photo-generated charge carriers, the CPR was characterized by varying applied AC voltage magnitude (*V*_ac_) under the dark and the greenish-yellow light conditions. As shown in the *C*_α_ response (Fig. 6a) under dark, there is no change in *C*_α_ irrespective of the *V*_ac_. In the presence of greenish-yellow light (Fig. 6b), with the increase in *V*_ac_, there is a significant increase in the capacitance. It means in this frequency regime, and at higher *V*_ac_, the charge carriers are getting modulated to the amplitude of the applied ac voltage and getting drifted towards the surface of the electrodes and making dielectric/nanocomposite less lossy. Whereas for smaller *V*_ac_ magnitude, the applied magnitude doesn’t impact the charge carriers, and they are within the bulk of the dielectric, making the dielectric medium lossy. Moreover, under dark conditions, the nanocomposite’s conductivity modulation is absent with varying *V*_ac_, as shown in Fig. 6a, owing to the absence of photo-generated carriers. The charge distribution of non-equilibrium photo-generated carriers under various bias conditions confirms the CPR mechanism as speculated. In addition to this, we characterized CPRs to observe any charge accumulation within devices after switching light as well as *V*_ac_ signal levels. The transient response of devices was monitored by illuminating them with the increasing greenish-yellow light intensity and subsequently with the decreasing intensity of the same magnitude. It can be observed from Fig. 6c, d that there is no significant effect of charge accumulation after switching, and the hysteresis is negligible. Hence there is no memory effect in the CPRs, which is essential for any tunable device. To summarize the operation mechanism of the CPR, on applying light illumination, there will be non-equilibrium charge carrier distribution within the bulk of the PFNC. This leads to a change in the pseudo-capacitance, *C*_α,_ of CPRs.

### Neuronal interface circuitry

The proposed CPR mimics the rod cells of the retina to develop a biomimetic eye. Light-sensitive devices can be used to build electronics that generate the change in spiking frequency, which is the functionality of the retina’s ganglion cells^6^. An array of these CPRs forms a receptive field of the retina through which a neural network for shape perception is feasible^9^. Thus, the interface circuit is highly needed to generate the spike train proportional to the light intensity and color. We first characterized the CPR device with the RC model using nonlinear least squares under illumination scenarios. The curve fitted model accurately model the frequency behavior of the CPR with a normalized root mean square error less than 3.3% (see the Supplementary Information S8 for more details on the fitted model and results).

**Fig. 7:**
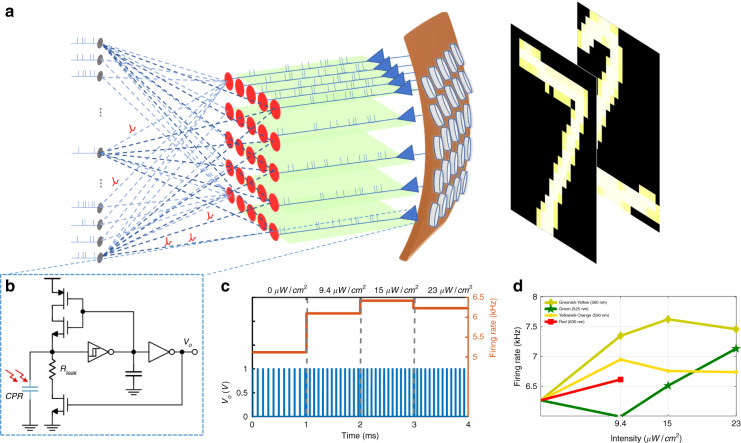
Neuronal interfacing and output response. **a** Spiking neural network processing the spike train, generated after seeing handwritten digits, **b** the schematic of low power CMOS circuit designed to interface with the CPR to generate the spike train. **c** The output spike train under greenish-yellow light and different illumination intensities, and **d** the firing rate under different light intensities and colors

An illustrative diagram showing the CPR connected to the sensing circuit followed by a spiking neural network to process the received information is depicted in Fig. 7a. The schematic of CMOS sensing circuit designed for interfacing the CPR to generate a spike train with a frequency proportional to incident light intensity is shown in Fig. 7b. By varying the incident light color and intensity, different spike frequencies are observed since each light and intensity have a different impact on the sensor. A sample of the spike train is shown in Fig. 7c, which was generated under the illumination of greenish-yellow light. Also, it is worth mentioning that the leakage resistor, *R*_leak,_ can be used to tune the spike rate. The higher the leakage resistor, the lower the firing rate. In order to evaluate the CPR, we tested different scenarios where the incident light intensity was varied for different colors, as depicted in Fig. 7d. Clearly, the firing rate (i.e., spike frequency) increases with the increase in light intensity, which is also observed for different colors, as shown in Fig. 7d. The greenish-yellow curve shows a better dynamic range (spanning from 6.7 to 8.2 kHz) compared to the other colors, which means better representation/ encoding of the incident light intensity. The generated spike trains can then be interfaced with any neuromorphic hardware such as Truenorth^66^ or Loihi^67^ to mimic the full functionality of the retina networks^68^.

### Unsupervised spiking neural network

The CPR with the interface circuit was simulated to interface a spiking neural network (SNN) to recognize handwritten digits. The pixels of the handwritten digits were encoded proportional to the light intensity, then converted to a spike train, as discussed in the previous subsection. This SNN is a single-layer network with 100 output neurons employing a winner-take-all (WTA) mechanism followed by a statistical output classifier. The network was trained with simplified spike-timing-dependent plasticity, STDP, with a leaky integrate-and-fire neuron model^69,70^. The details of the neuron, synaptic conductance, and STDP models are discussed in detail in S9 in the supplementary materials. In this work, we consider STDP training, which is more brain-plausible. We used the trained model, without retraining, to evaluate the performance of CPRs and the interface circuit where the feature maps have already learned in the initial training, and the is no need to retrain the network. The network shows 70.98 and 72.05% recognition accuracy for greenish-yellow and violet lights, respectively. The accuracy loss is due to the nonlinearity of the simple interface circuit. Further optimized circuits can be used to enhance the overall linearity and sensitivity, which would be reflected in the SNN accuracy to bridge the 15% drop. The state-of-the-art training models have shown 87.25% accuracy in recognizing the MNIST dataset^70,71^. The low recognition accuracy is due to the shallowness of the used network and that unsupervised training is used. The accuracy can reach 95% by increasing the number of neurons to 1500^71^, or by applying state-of-art supervised learning methods such as SuperSpike^72^ or DECOLLE^73^.

## Discussion

We demonstrated the facile fabrication of tunable and flexible capacitive photoreceptors using a hybrid nanocomposite of metal halide perovskite (CH_3_NH_3_PbBr_3_) and ferroelectric polymer (PVDF-TrFE-CFE). The purity and morphology of the perovskite nanocrystals are undisturbed in the nanocomposite and exhibited excellent light absorption properties. The characterized CPR’s capacitance is tunable and reproducible with the light intensity and frequency independent in the 1−100 kHz regime at each light intensity. The mechanism of the CPR was studied through optoelectric characterizations. The stable and non-degradable performance of metal halide perovskite nanocrystals-based devices is observed for 129-weeks. The highly sensitive CPR mimics the rod cells of the retina. The developed CPR is interfaced with simple circuitry and a simple neural network to demonstrate its functionality as a biomimetic retina. The demonstrated system has massive potential in developing artificial retina networks and biomimetic eyes for perceptive intelligence applications. In addition, such CPRs find their unique place in developing photosensitive actuators for robotic applications, and these can also be explored as optoelectronic devices for optical communications.

## Materials and methods

### Fabrication of the CPR

The solution of the nanocomposite was prepared in two steps. At first, 0.5 M CH_3_NH_3_PbBr_3_ (MAPbBr_3_) solution^39^ was prepared from the commercial methyl ammonium bromide (CH_3_NH_3_Br) and lead bromide (PbBr_2_), the mixture was added to the N, N Dimethylformamide (DMF) solvent and ultra-sonicated for 24 h. In the second step, 100 mg of commercial ferroelectric terpolymer (FP) PVDF-TrFE-CFE was stirred constantly in 1 ml of DMF solvent for 24 h. The final PFNC solution was prepared by mixing 1 ml of the FP solution with the 1 ml of MAPbBr_3_ solution. The mixture was stirred constantly for 24 h to obtain a homogenous PFNC solution. We have employed an aluminum-coated Kapton Polyimide sheet that serves as the flexible substrates for flexible CPR. 300 μL of PFNC solution was drop-casted on the square-shaped (2 × 2 cm^2^) Al coated polyimide sheet pasted on the carrier substrate. The substrate was heated under vacuum for 3 h at 90 °C for solvent evaporation. 120 nm thick transparent indium tin oxide (ITO) was RF sputtered on the PFNC thin-film using a shadow mask for the top electrode (3 mm circular form). It was observed that by adding the MAPbBr_3_ solution as filler in the FP solution, the phase angle could be tuned to frequency-independent as reported for semiconducting fillers in the FP solution^55^. The final weight percentage of FP and MAPbBr_3_ solutions of PFNC was optimized to get a frequency-independent phase angle under the dark condition was achieved. A similar process can also be followed on a rigid metal-coated Silicon substrate, and flexibility can be achieved through the soft-backside etch of the silicon substrate^74,75^. The PVDF-CrFE-TFE powder was purchased from Piezotech. DMF (N, N-dimethylformamide, 99.8%) solvent, PbBr_2_ (99%), CH_3_NH_3_Br (98%) powders were purchased from Sigma-Aldrich.

### Imaging and characterization

A FEI- Quanta 600 FEG scanning electron microscope operated at 10 kV was used to capture the cross-sectional image of the perovskite ferroelectric nanocomposite (PFNC). The CPR sample was dipped in liquid nitrogen to have a sharp cross-section cut for imaging. The sample was coated with a layer of 3 nm of iridium. The transmission electron microscope imaging was performed using FEI-Tecnai Spirit Biotwin. The TEM sample was prepared by drop-casting the PFNC solution on the copper grid. The UV–visible absorbance of the PFNC was measured using a Perkin Elmer’s UV−VIS spectrophotometer lambda 950, which was equipped with an integrated sphere accessory to measure reflectance/absorbance of thin-films. The absorbance was measured in the range of 200–850 nm at a scanning speed of 1 nm/s. The X-ray diffraction studies on both PVDF and PFNC were performed using the XRD Bruker D2 Phaser instrument. Samples for study were exposed to the X-ray source for 4 min. The XRD measurements were studied on PFNC/Au/Si and FP/Au/Si samples. Photoluminescence analysis was performed with the WITec Apyron spectrometer, and samples are excited with laser (λ = 473 nm).

### Electrical characterization

The electrical characterization was performed using an Agilent 4980A LCR meter. In all the experiments, unless otherwise mentioned, the AC voltage bias is 0.1 V. The LEDs were biased using a voltage source. A LabVIEW data acquisition software was designed to acquire the data used in all the experiments.

### Circuit model identification

Finding a circuit model for the fabricated CPR is essential to be incorporated in the retina simulators and to design suitable interface circuits. In order to characterize our device, a shunt capacitor, and a shunt conductance in addition to parallel RC branches, inspired from Fig. 3f was used to model the leakage, electrode capacitances, and dielectric, respectively. Nonlinear least-squares fitting function in MATLAB was used to model identification where the loss function is defined to minimize *L*^2^ norm of the relative error of the real and the imaginary parts of the admittance over the frequency range. The normalized root mean square error of the extracted model is less than 3.37% in the worst case.

## Supplementary information

A Flexible Capacitive Photoreceptor for the Biomimetic Retina
