# Supplementary material for: A flexible capacitive photoreceptor for the biomimetic retina
Source: Light Sci Appl. 2022 Jan 1;11:3. doi: 10.1038/s41377-021-00686-4 (PMC8720312; doi:10.1038/s41377-021-00686-4)
Supplement: Supplementary file 1 — A Flexible Capacitive Photoreceptor for the Biomimetic Retina [file 41377_2021_686_MOESM1_ESM.docx]

Supplementary Information

**A Flexible Capacitive Photoreceptor for the Biomimetic Retina**

*Mani Teja Vijjapu*^1^*, Mohammed E. Fouda,*^2,3^ *Agamyrat Agambayev,*^1,4^ *Chun Hong Kang,*^5^ *Chun-Ho Lin,^5^ Boon S. Ooi,* ^5^ *Jr-Hau He,^5,6^ Ahmed M. Eltawil,*^2,3^ *and Khaled N. Salama*^1^*^*^*

^1^Sensors lab, Advanced Membranes and Porous Materials Center, Computer, Electrical and Mathematical Science and Engineering Division, King Abdullah University of Science and Technology (KAUST), Thuwal, 23955-6900, Kingdom of Saudi Arabia

^2^ Communication and Computing Systems Lab, Computer, Electrical and Mathematical Science and Engineering Division, King Abdullah University of Science and Technology (KAUST), Thuwal, 23955-6900, Kingdom of Saudi Arabia

^3^Department of Electrical Engineering and Computer Science, University of California-Irvine, Irvine, CA 92612, USA

^4^Department of Electrical, Computer and Energy Engineering, Arizona State University, Tempe, Arizona, USA

^5^Computer, Electrical and Mathematical Science and Engineering Division, King Abdullah University of Science and Technology (KAUST), Thuwal, 23955-6900, Kingdom of Saudi Arabia

^6^Department of Materials Science and Engineering, City University of Hong Kong, Hong Kong SAR, China

**S1: The X-ray diffraction spectra**

The X-ray diffraction (XRD) studies were performed to study the crystalline nature of perovskite (CH_3_NH_3_Br_2_) nanocrystals after the composite preparation. The PVDF-TrFE-CFE composite without perovskite nanocrystals (FC/Au) and with nanocrystals (PFNC/Au) were drop-casted on rigid Au/SiO_2_/Si substrate for the XRD analysis. The spectra are shown in Figure. S1 indicates that CH_3_NH_3_Br_2_ nanocrystals are undisturbed after composite preparation.


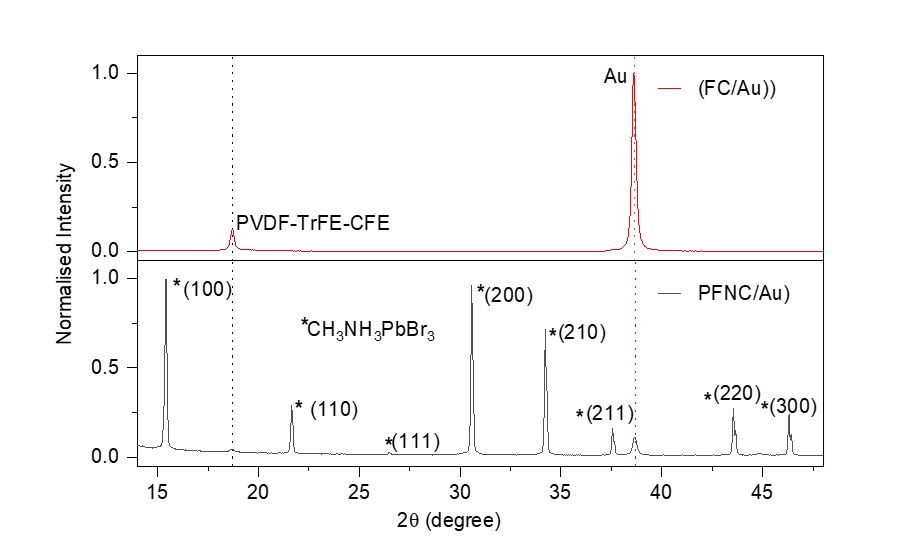


Figure. S1. The X-ray diffraction patterns of the ferroelectric composites without perovskite (FC) and with perovskites (PFNC) show the purity of the perovskite crystals in the composite.

**S2: Setup for photoresponse characterization**

Setup was made to obtain a square and homogenized beam for commercial LEDs to investigate the device response, as shown in Figure. S2a. The emission spectra of LEDs are shown in Figure. S2b **(violet (~ *λ_pe_*_ak_=403 nm), blue (~ *λ_peak_*=457 nm), green (~ *λ_peak_*=525 nm), greenish-yellow (~ *λ_peak_*=560 nm), yellowish-orange (~ *λ_peak_*=590 nm) and red (~ *λ_peak_*=630 nm).** The biasing conditions of LEDs were optimized to get uniform intensities in all the wavelength regimes. The capacitive photoreceptors (CPR) were electrically characterized under the controlled light conditions to study their optoelectronic properties.


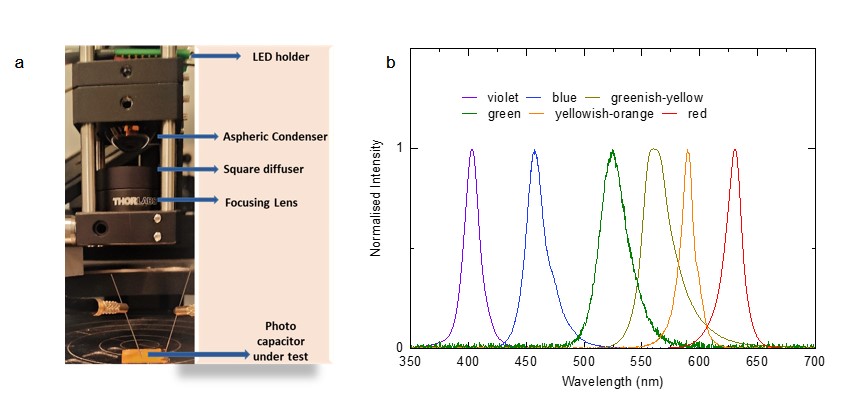


Figure. S2. a) The digital photograph of the optical setup and the corresponding parts are labeled. b) Emission spectra of LEDs used for the capacitive photoreceptors characterization

**S3: Response of capacitive photoreceptors under violet LED**

Pseudo-Capacitance is defined as

$$C_{\alpha}=\frac{1}{\left| Z_{in} \right|\omega^{\alpha}}$$

where $\alpha=\frac{2}{\pi}tan^{-1}\left( \frac{Z^{''}}{Z^{'}} \right)$, and $Z^{'}$ and $Z^{''}$are real and imaginary parts of the device impedance, $Z_{in}$. The pseudo-capacitance of CPRs under violet at low and higher intensities is shown in Figure. S3. The CPRs exhibited a similar response as under greenish-yellow light, but the extent of variation is less in the case of violet at the same intensity. This is due to the absorbance of the composite in corresponding wavelength regimes.


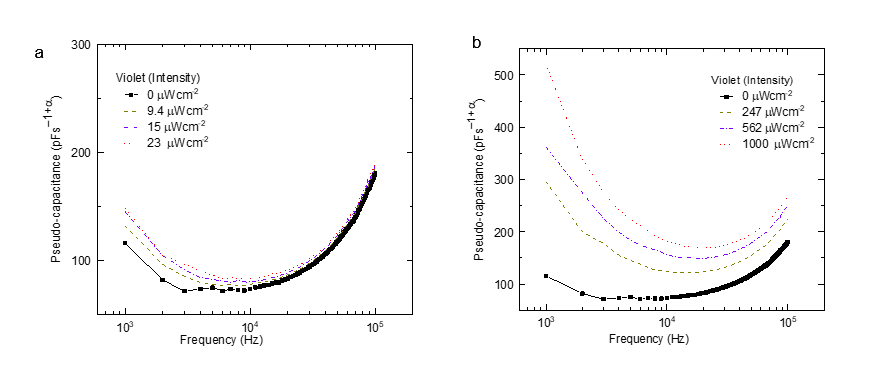


Figure. S3. The capacitance of capacitive photoreceptors under the illumination of violet a) lower intensity b) higher intensity

**S4: Nyquist impedance plot under violet and greenish-yellow LEDs**

The Nyquist impedance plots showing the frequency-independent capacitive behavior of CPRs under various light conditions are shown in Figure. S4.


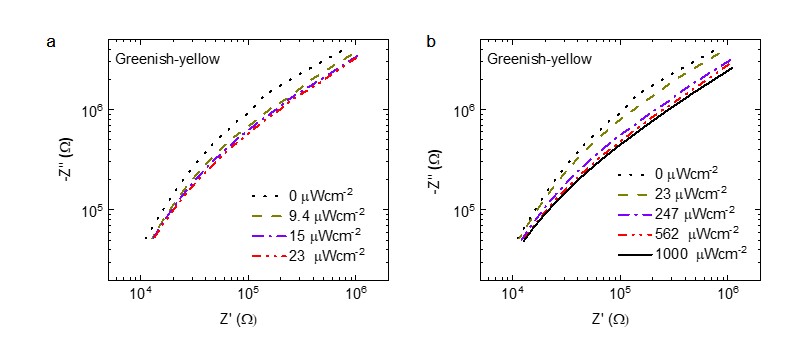


Figure. S4. The Nyquist impedance plot showing the frequency-independent capacitive behavior under the illumination of a) greenish-yellow b) violet LEDs

**S5: Response of capacitive photoreceptor under a greenish-yellow LED after 100 weeks**

The response of the CPR under the most sensitive greenish-yellow regime was measured after 100 weeks of the device fabrication. These devices were stored at room temperature and ambient air conditions (23 ^o^C and 40 %R_H_). The response shows the CPR is still sensitive to the light and exhibits frequency-independent capacitive behavior. This response also indicates that the perovskite nanocrystals are highly stable due to the encapsulation in the polymer without any degradation due to the external environment.


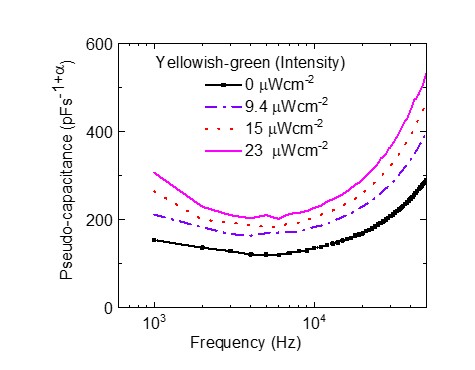


Figure. S5. The response of capacitive photoreceptors of the device measured after 100 weeks of fabrication under the illumination of greenish-yellow led (560 nm)

**S6: The long-time measurement and transient response of capacitive photoreceptors under dark and greenish-yellow light**

The transient, as well as long-time measurements, were performed on the CPRs in different light conditions to understand the electrical stability. Figure. S6a indicates that in the dark condition, the capacitance is constant. When there is light, the capacitance initially overshoots and stabilizes to a constant value within a second. Moreover, under continuous illumination, for more than 120 min, the capacitance is stable without any variation in its value, and it indicates that there is negligible charge carrier accumulation in the device.

To understand the response time, devices were excited with modulating LED light (LED ON time 10 s and OFF time 5 s), and the capacitance is measured for every 0.24 s using an LCR meter. As shown in Figure. S6b, the devices reach a stable value in ~ 0.8 s when LED is ON. Whereas the devices reach the baseline in 0.92 s, when LED is OFF, as shown in Figure. S6c.


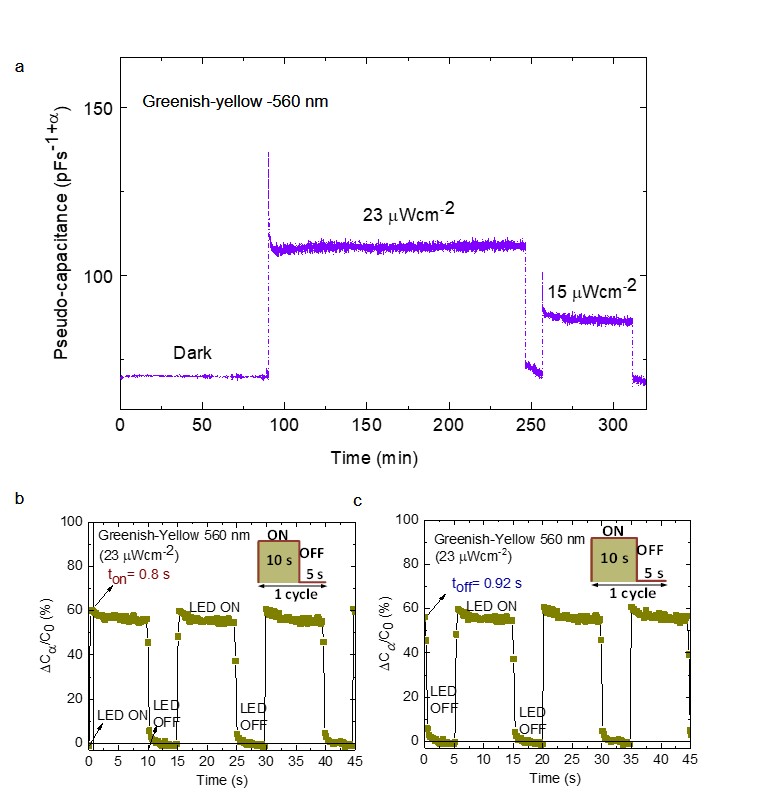


Figure. S6. a) The response of the capacitive photoreceptor showing stability under dark for a long time. Under the illumination (greenish-yellow), the capacitor overshoots and stabilizes to a constant capacitance value. b), c) Responses of CPR devices measured with faster sampling rate 10 ms/sample using LCR meter. The arrow indicates the time taken to reach the steady-state (*t_on_* and *t_off_*).

**S7: The setup to test the capacitive photoreceptor after bending**

The following setup (Figure. S7) was used to measure the CPR response before and after bending. The flexible CPR was placed on the hollow cylinder with a bending radius of 1 cm. The response was measured under the illumination of greenish-yellow led.


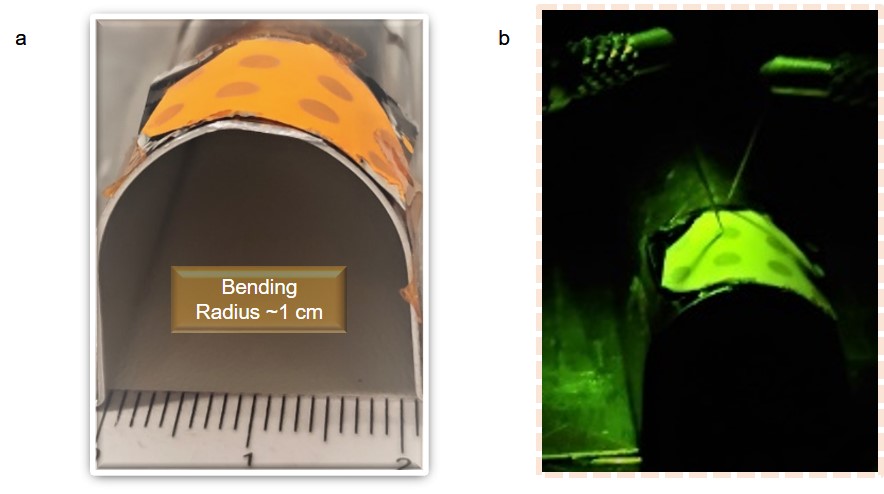


Figure. S7. a) The setup used to measure the photoresponsive properties of the capacitive photoreceptor after bending (bending radius =1 cm). b) The digital photograph of the bent CPR under greenish-yellow illumination.

# S8: Device parameter Identification

Finding a circuit model for the device is essential to be incorporated in the retina simulators to design suitable interface circuits. In order to characterize our device, an *RC* network was used, as shown in Figure. S8a, which is widely used to realize fractional-order capacitors. The input admittance of the network is defined as follows:

$$Y_{in}=\frac{1}{R_{p}}+sC_{p}+\sum_{k=1}^{N} \frac{sC_{k}}{sC_{k}R_{k}+1}$$

where $G_{p}$ and $C_{p}$ are shunt conductance and capacitance, $R_{k}$and $C_{k}$ are the resistance and capacitance of the $k^{th}$ branch, and $N$ is the number of parallel branches.

In order to extract values of model parameters, we used the least-squares fitting function in MATLAB, *lsqcurvefit,* where the loss function is defined to minimize $L^{2}$ norm of the relative error of the real and the imaginary parts of the admittance over the frequency range. The relative error was used to avoid the biased solution, which might result from unequal ranges of the real and imaginary parts like in our device where the imaginary part is 30x higher than the real part of the impedance. The loss function is defined as follows:

$$\min_{x} \sum_{i}^{M} \left( \frac{Y_{in}^{'}\left( x,f_{i} \right)-Y_{meas}^{'}\left( f_{i} \right)}{Y_{meas}^{'}\left( f_{i} \right)} \right)^{2}+\left( \frac{Y_{in}^{''}\left( x,f_{i} \right)-Y_{meas}^{''}\left( f_{i} \right)}{Y_{meas}^{''}\left( f_{i} \right)} \right)^{2}$$

where $x$ is the search vector and is defined as $\left[ R_{p}, C_{p},R_{1},C_{1}, \ldots, R_{N},C_{N} \right]$, $M$ is the number of the frequency points, $Y_{in}^{'}$ and $Y_{in}^{''}$ are the real and imaginary parts of the admittance model, and $Y_{in}^{'}$ and $Y_{in}^{''}$ are the real and imaginary parts of the measured admittance.

The proposed parameter identification algorithm was used to extract the device parameters with *N*=9. Figure. S8b-g, show the curved model results on top of the measured data showing a good matching. For better visibility of the results, we plotted only some selected cases. Table S1 shows the normalized root mean square error results of the curve fitted model with at most 3.37% error.

Table S1. Normalized root mean square error of the fitted *RC* circuit model with 1.55% for the dark condition.

| $\mu wcm^{-2}$ | $9.4$ | $15$ | $23$ | $247$ | $562$ | $1000$ |
| --- | --- | --- | --- | --- | --- | --- |
| Greenish-yellow | 0.814% | 0.61% | 0.56% |  |  |  |
| Violet | 0.626% | 0.394% | 0.875% | 0.927% | 0.85% | 0.8479% |
| Green | 2.29% | 3.37% | 2.64% |  |  |  |
| Blue | 1.84% | 2.56% | 1.58% |  |  |  |
| Yellowish-orange | 1.678% | 2.26% | 1.52% |  |  |  |
| Red | 1.49% |  |  |  |  |  |


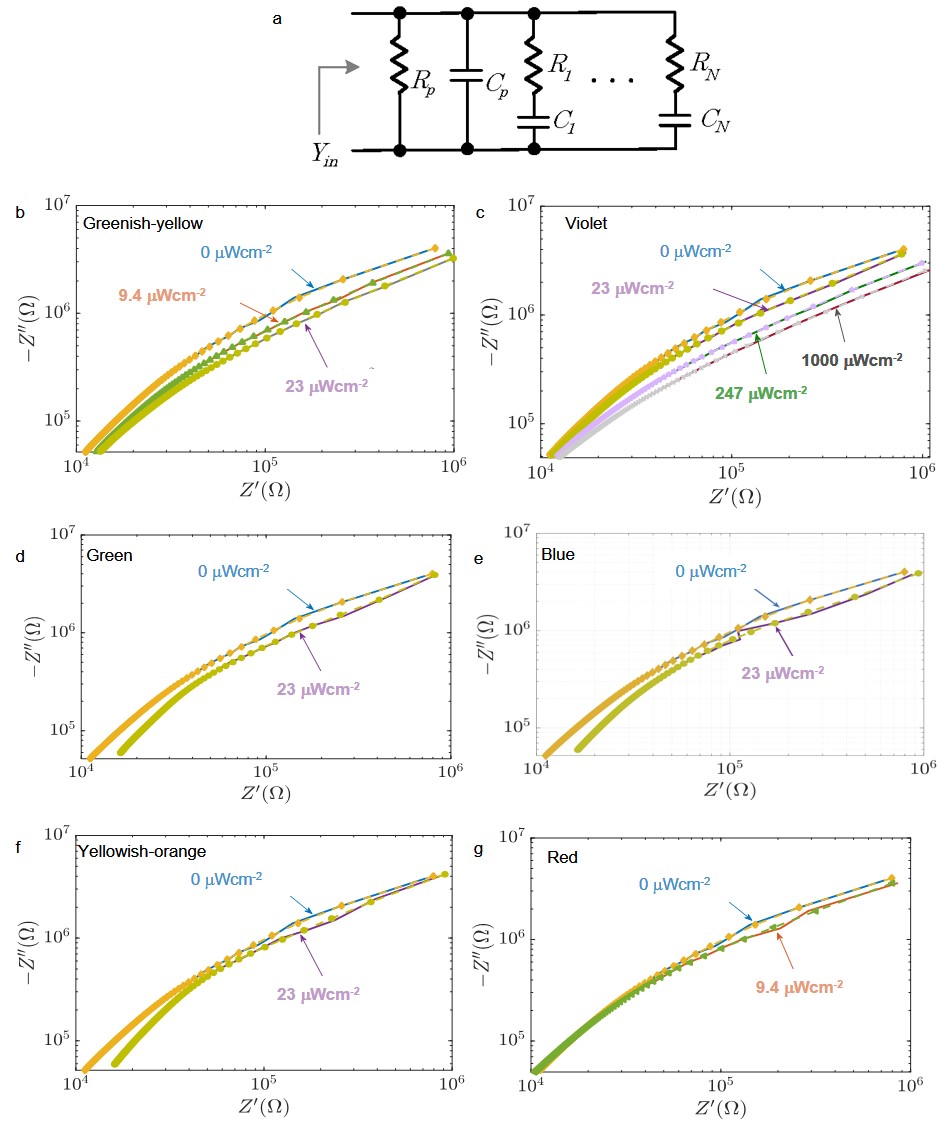


Figure. S8. a) *RC* circuit Model, and b-g) Nyquist impedance plots of the device model (dashed-squares lines) on top of the measured results (solid lines) for different colors and illumination intensities.

**Power consumption of CPR:**

Table S2. summarizes the proposed CPR sensor’s power consumption compared with the reported photodetectors. The CPR power consumption is estimated during its operation within the interface circuit. In order to have a fair comparison against other photodetectors, we normalized the power consumption of reported photodetectors per 1 μWcm^-2^.

Table S2. Power consumption comparison of proposed CPR with the reported photodetectors used to develop a biomimetic eye.

| Sl.  No. | Photodetector material | Power Consumption | | Reference |
| --- | --- | --- | --- | --- |
|  |  | In light  (per 1 μWcm^-2^) | Dark |  |
| 1. | Perovskite nano wires (FAPbI_3_) | 8.1 pW | 135 nW | ^1^ |
| 2. | Si Photodetector | N/A | 50 nW | ^2^ |
| 3. | Micro cavity perovskite (MAPbI_3_) | 50 pW | 30.6 nW | ^3^ |
| 4. | MoS_2_ based Phototransistor | 0.75 pW | 2.40 nW | ^4^ |
| 5. | 3D Printed polymer | 2 pW | 50 nW | ^5^ |
| 6. | CPR | 0.6957 pW | 9.6 pW | Ours |

**S9. Spiking Neural Network Model**

**S9a. Neuron Model**

In this work, a leaky integrate-and-fire (LIF) model was employed. It is widely used in neuromorphic systems due to its low computational complexity and effectiveness in capturing information processing features.^6^ The LIF model consists of a first-order linear differential equation which defines the dynamics of membrane potential, $v$, and is defined as

$$\tau_{m}\frac{dv}{dt}=\left( v_{r}-v \right)-g_{e}\left( v-E_{exc} \right)-g_{i}\left( v-E_{inh} \right)$$

where $\tau_{m}$*,* $v_{r}$*,* $g_{l}$*,* $g_{e}$ and $g_{i}$ are the time constant, the resting membrane potential, the leak conductance, the excitatory conductance associated with an excitatory channel, and the conductance associated with an inhibitory channel, respectively. $E_{exc}$ $E_{inh}$*,* and $v_{r}$ are the excitatory, the inhibitory, and the resting potentials of the channel, respectively. The model generates a spike if the membrane potential reaches the threshed potential, $v_{th}$. And, the synaptic conductance with time-varying dynamics is defined as follows,^7^

$$\tau_{g}\frac{dg}{dt}=-g+\sum_{j} w_{ij}\delta(t-t_{j}^{f})$$

where $g$ and $\tau_{g}$are the conductance and the time constant, respectively. $w_{ij}$ is the synaptic weight connecting the *j^th^* presynaptic neuron to the *i^th^* postsynaptic neuron *i*, and $t_{j}^{f}$is the firing time of the *j^th^* neuron *j*.

**S9b. Learning model**

In this work, we utilize a well-known brain-inspired mechanism for unsupervised learning called

Spike-timing-dependent plasticity (STDP) relates synaptic plasticity to the timing difference between the presynaptic and postsynaptic spikes. We employed a simplified STDP to update synaptic weights given as^8^

$$\Delta w_{ij}=\left\{ \begin{aligned} \mu_{pre}w_{ij}\left( 1-w_{ij} \right), \mathrm{if} t_{j}-t_{i}<0, \\ \mu_{post}w_{ij}\left( 1-w_{ij} \right), \mathrm{if} t_{j}-t_{i}>0. \end{aligned} \right.$$

where $\mu_{pre}$ and $\mu_{post}$ are the learning rates.

Table S3. Model parameters used in the simulation.

| Model parameters | Description | Value |
| --- | --- | --- |
| $\tau_{m}, \tau_{g}$ | Time constants for the LIF model | 10 ms, 30 ms |
| $v_{r}, v_{th}, E_{exc}, E_{inh}$ | Potential constants for LIF model | 0, 60 mV, 0, -100 mV |
| $\mu_{pre}, \mu_{post}$ | Learning rates for STDP model | -0.002, 0.02 |

**S9c. SNN model and Training**

In this work, we considered a single-layer SNN, shown in Figure S9, followed by a processing layer. The SNN layer size is $784\times100,$ where 784 is the input image size ($28\times28$) for the Modified National Institute of Standards and Technology (MNIST) dataset. The output of each spiking circuit is connected to the synapses of the first layer in the network. The second layer is used to implements a winner-take-all (WTA) mechanism, which consists of 100 excitatory neurons connected to 100 inhibitory neurons in a one-to-one configuration. Each inhibitory neuron is connected to all the excitatory neurons except the one that receives the spikes from, as shown in Figure S9. WTA imposes lateral inhibition on excitatory neurons and hence competitions for learning input features. The processing layer is a simple classification scheme that classifies the outputs based on the firing activity of excitatory neurons. Once the STDP training is over, the excitatory neurons are assigned labels by matching the firing neurons with the input images. In MNIST cases, the excitatory neurons are divided into 10 groups since there are 10 labels in MNIST dataset. After the training and labeling of the neurons, the network is ready to be used for inference mode where the STDP is deactivated.


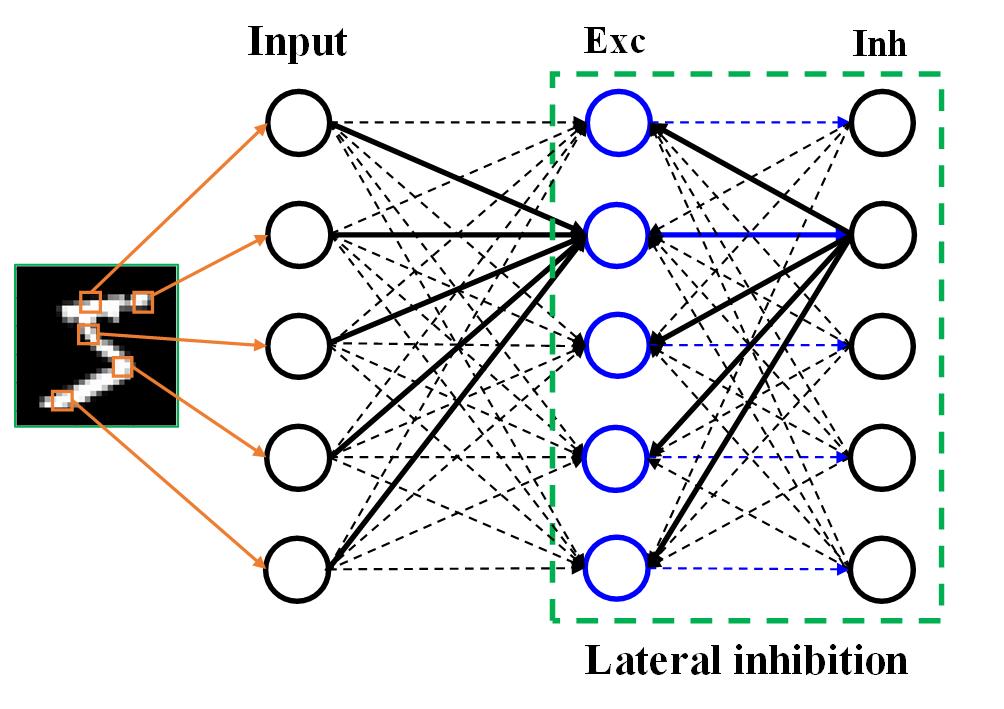


**Figure S9** spiking neural network architecture.

Under the aforementioned setup, the state of art recognition accuracy is 87.25%.^7, 9^ The low recognition accuracy is due to the shallowness of the used network and the unsupervised training of the network. The accuracy can reach 95% by increasing the number of neurons to 1500,^7^ or by applying state-of-art supervised learning methods such as SuperSpike^10^ or DECOLLE.^11^ In this work, we consider STDP training which is more brain plausible.

In this work, we used the trained network to recognize the spike trains generated from CPR and sensing circuits, which is known as transfer learning, without retraining the neural network. Since the network already has the feature maps of the dataset, hence there is no need for retraining. In our software level simulator, we modeled the firing rate versus the light intensity with a quadratic function and generated spike trains with a frequency proportional to the light intensity based on that fitted quadratic function. The fitted firing rates for greenish-yellow and violet are given as follows:

$f_{r\_gy} = 5860 + 13.9263 P - 0.0368 P^{2}$, and

$$f_{r\_v}= 5916 + 14.9686 P - 0.035 P^{2},$$

Respectively, where *P* is the pixel value and enclosed in [0, 255]. The recognition accuracy is found to be 70.98 % and 72.05% for greenish-yellow and violet lights, respectively. The accuracy loss is due to the nonlinearity of the simple interface circuit and the limited dynamic range. In this work, we considered a simple circuit and simple network to show the functionality of the overall system.

**References:**

1. Gu, L. et al. A biomimetic eye with a hemispherical perovskite nanowire array retina. *Nature* **581**, 278-282 (2020).

2. Ko, H.C. et al. A hemispherical electronic eye camera based on compressible silicon optoelectronics. *Nature* **454**, 748-753 (2008).

3. Tsai, W.-L. et al. Band Tunable Microcavity Perovskite Artificial Human Photoreceptors. *Advanced Materials* **31**, 1900231 (2019).

4. Choi, C. et al. Human eye-inspired soft optoelectronic device using high-density MoS2-graphene curved image sensor array. *Nature Communications* **8**, 1664 (2017).

5. Park, S.H. et al. 3D Printed Polymer Photodetectors. *Advanced Materials* **30**, 1803980 (2018).

6. Burkitt, A.N. A Review of the Integrate-and-fire Neuron Model: I. Homogeneous Synaptic Input. *Biological Cybernetics* **95**, 1-19 (2006).

7. Diehl, P. & Cook, M. Unsupervised learning of digit recognition using spike-timing-dependent plasticity. *Frontiers in Computational Neuroscience* **9** (2015).

8. Masquelier, T. & Thorpe, S.J. Unsupervised learning of visual features through spike timing dependent plasticity. *PLoS Comput Biol* **3**, e31 (2007).

9. Guo, W., Fouda, M.E., Yantir, H.E., Eltawil, A.M. & Salama, K.N. Unsupervised Adaptive Weight Pruning for Energy-Efficient Neuromorphic Systems. *Frontiers in Neuroscience* **14** (2020).

10. Zenke, F. & Ganguli, S. SuperSpike: Supervised Learning in Multilayer Spiking Neural Networks. *Neural Computation* **30**, 1514-1541 (2018).

11. Kaiser, J., Mostafa, H. & Neftci, E. Synaptic Plasticity Dynamics for Deep Continuous Local Learning (DECOLLE). *Frontiers in Neuroscience* **14** (2020).
